# Prognostic value of early EEG abnormalities in severe stroke patients requiring mechanical ventilation: a pre-planned analysis of the SPICE prospective multicenter study

**DOI:** 10.1186/s13054-024-04957-5

**Published:** 2024-05-23

**Authors:** Sarah Benghanem, Nathalie Kubis, Etienne Gayat, Ambre Loiodice, Estelle Pruvost-Robieux, Tarek Sharshar, Arnaud Foucrier, Samy Figueiredo, Viviane Bouilleret, Etienne De Montmollin, François Bagate, Jean-Pascal Lefaucheur, Bertrand Guidet, Emmanuelle Appartis, Alain Cariou, Olivier Varnet, Paul Henri Jost, Bruno Megarbane, Vincent Degos, Loic Le Guennec, Lionel Naccache, Stephane Legriel, France Woimant, Charles Gregoire, David Cortier, Isabelle Crassard, Jean-François Timsit, Mikael Mazighi, Romain Sonneville, and the SPICE investigators, Tiare Ader, Eric Barré, Hélène Bout, Perrine Boursin, Eric Bodiguel, Damien Bresson, Omar Ben Hadj Salem, Alain Combes, Anne Chrisment, Magalie Collet, Jacque Duranteau, Sophie Crozier, Daniel da Silva, Amexandre Demoule, Maxens Decavele, Eric Delpierre, Jean Luc Diehl, Martin Dres, Frédéric Faugeras, Marie-Céline Fournier, Tobias Gauss, Coralie Gernez, Guillaume Geri, Dominique Hurel, Matthieu Jamme, Laurence Josse, Igor Jurcisin, Lionel Kerhuel, Catherine Lamy, Fariza Lamara, Aymeric Lancelot, Bertrand Lapergue, Christophe Lenclud, Mathilde Lermuzeaux, Eric Magalhaes, Eric Mariotte, Isabelle Malissin, Alain Maldjian, Nathalie Marin, Jérôme Martin, Thibault Martinez, Armand Mekontso Dessap, Mehran Monchi, Giulia Naim, Hervé Outin, David Osman, Gregory Papin, Pierre Pasquier, Claire Pichereau, Matthieu Pissot, Keyvan Razazi, Danielle Reuter, Christian Richard, Stephane Ruckly, Damien Roux, Caroline Schimpf, Quentin Staiquly, Jérôme Servan, Sebastien Tanaka, Laurie-Anne Thion, Karim Toumert, Widad Traki, Marc Tran, Philippe Vassel, Bernard Vigué, Daniel Zafimahazo, Jonathan Zarka

**Affiliations:** 1 AP-HP.Centre, Medical ICU, Cochin Hospital, Paris, France; 2 University Paris Cité, Medical School, Paris, France; 3 INSERM UMR 1266, Institut de Psychiatrie et Neurosciences de Paris-IPNP, Paris, France; 4 APHP.Nord, Clinical Physiology Department, UMRS_1144, Université Paris Cite, Paris, France; 5 APHP.Nord, Department of Anesthesiology and Critical Care, DMU Parabol, Université Paris Cite, Paris, France; 6 Department of Biostatistics, ICUREsearch, Paris, France; 7 Neurophysiology and Epileptology Department, GHU Psychiatry & Neurosciences, Sainte Anne, Paris, France; 8 Department of Neuroanesthesiology and Intensive Care, Sainte Anne Hospital, Paris, France; 9 APHP, Department of Anesthesiology and Critical Care, Beaujon University Hospital, Clichy, France; 10 APHP, Department of Anesthesiology and Critical Care, Bicêtre University Hospitals, Le Kremlin Bicêtre, France; 11 Neurophysiology and Epileptology Department, Bicêtre University Hospitals, Le Kremlin Bicêtre, France; 12 Department of Intensive Care Medicine, Delafontaine Hospital, Saint-Denis, France; 13 APHP, Department of Intensive Care Medicine, Henri Mondor University Hospital and Université de Paris Est Créteil, Créteil, France; 14 APHP, Neurophysiology Department, Henri Mondor University Hospital, Créteil, France; 15 APHP, Department of Intensive Care Medicine, Saint Antoine University Hospital, Paris, France; 16 Neurophysiology Department, Saint Antoine University Hospital, Paris, France; 17 APHP, Department of Physiology, Bichat-Claude Bernard University Hospital, 75018 Paris, France; 18 APHP, Department of Anesthesiology and Intensive Care, Henri Mondor Hospital, Creteil, France; 19 APHP, Medical ICU, Lariboisiere Hospital, Paris, France; 20 APHP, Department of Anesthesiology and Neurointensive Care, Pitié Salpétrière Hospital, Paris, France; 21 APHP, Medical ICU, Pitié Salpétrière Hospital, Paris, France; 22 APHP, Department of Physiology, Pitié Salpétrière Hospital, Paris, France; 23 Medical ICU, Le Chesnay Hospital, Versailles, France; 24 Agence Régionale de Santé Ile-de-France, Paris, France; 25 Department of Intensive Care, Rothschild Hospital Foundation, Paris, France; 26 Department of Intensive Care, Foch Hospital, Paris, France; 27 APHP, Department of Intensive Care Medicine, Bichat-Claude Bernard University Hospital, 46 rue Henri Huchard, 75018 Paris, France; 28 Université Paris Cité, INSERM UMR 1137, IAME, Paris, France; 29 APHP Nord, Department of Neurology, Lariboisière University Hospital, Department of Interventional Neuroradiology, Fondation Rothschild Hospital, FHU Neurovasc, Paris, France; 30 Université Paris Cité, INSERM UMR 1144, Paris, France

**Keywords:** Severe stroke, Electroencephalogram, EEG reactivity, Prognostication, Intensive care

## Abstract

**Introduction:**

Prognostication of outcome in severe stroke patients necessitating invasive mechanical ventilation poses significant challenges. The objective of this study was to assess the prognostic significance and prevalence of early electroencephalogram (EEG) abnormalities in adult stroke patients receiving mechanical ventilation.

**Methods:**

This study is a pre-planned ancillary investigation within the prospective multicenter SPICE cohort study (2017–2019), conducted in 33 intensive care units (ICUs) in the Paris area, France. We included adult stroke patients requiring invasive mechanical ventilation, who underwent at least one intermittent EEG examination during their ICU stay. The primary endpoint was the functional neurological outcome at one year, determined using the modified Rankin scale (mRS), and dichotomized as unfavorable (mRS 4–6, indicating severe disability or death) or favorable (mRS 0–3). Multivariable regression analyses were employed to identify EEG abnormalities associated with functional outcomes.

**Results:**

Of the 364 patients enrolled in the SPICE study, 153 patients (49 ischemic strokes, 52 intracranial hemorrhages, and 52 subarachnoid hemorrhages) underwent at least one EEG at a median time of 4 (interquartile range 2–7) days post-stroke. Rates of diffuse slowing (70% vs. 63%, *p* = 0.37), focal slowing (38% vs. 32%, *p* = 0.15), periodic discharges (2.3% vs. 3.7%, *p* = 0.9), and electrographic seizures (4.5% vs. 3.7%, *p* = 0.4) were comparable between patients with unfavorable and favorable outcomes. Following adjustment for potential confounders, an unreactive EEG background to auditory and pain stimulations (OR 6.02, 95% CI 2.27–15.99) was independently associated with unfavorable outcomes. An unreactive EEG predicted unfavorable outcome with a specificity of 48% (95% CI 40–56), sensitivity of 79% (95% CI 72–85), and positive predictive value (PPV) of 74% (95% CI 67–81). Conversely, a benign EEG (defined as continuous and reactive background activity without seizure, periodic discharges, triphasic waves, or burst suppression) predicted favorable outcome with a specificity of 89% (95% CI 84–94), and a sensitivity of 37% (95% CI 30–45).

**Conclusion:**

The absence of EEG reactivity independently predicts unfavorable outcomes at one year in severe stroke patients requiring mechanical ventilation in the ICU, although its prognostic value remains limited. Conversely, a benign EEG pattern was associated with a favorable outcome.

**Supplementary Information:**

The online version contains supplementary material available at 10.1186/s13054-024-04957-5.

## Introduction

Stroke is one of the most common acute neurological disorders, and is associated with a high rate of long-term disability and mortality [1]. Severe stroke patients may require invasive mechanical ventilation (MV) in an intensive care unit (ICU) mainly because of impairment of consciousness, seizures, neurosurgical and neuroradiological procedures, and/or respiratory complications [2]. Outcome of patients with severe stroke requiring MV remains poor, with mortality rates of ± 50% and good functional outcome at one year in 33% of cases [3]. Although well described in patients with hypoxic ischemic brain injury (HIBI) after cardiac arrest (CA) and traumatic brain injury (TBI), early prognostication markers remain poorly studied in severe stroke patients [4, 5]. Common predictors mainly include clinical severity scales such as the Glasgow Coma Scale (GCS) and National Institutes of Health Stroke Scale (NIHSS), and neuroimaging features at stroke onset [6, 7].

Recently, there has been a growing interest in electroencephalogram (EEG) monitoring in brain injured patients in the ICU [8]. EEG is characterized by a high temporal resolution and could thus detect secondary brain injury in real time, leading to important changes in management, including brain imaging and therapeutic interventions (i.e., neuroradiological or neurosurgical treatment). This tool has also been recognized as a robust prognostic marker after CA, both for unfavorable neurological outcome prediction when “highly malignant patterns” are present (i.e., suppression with or without periodic discharges and burst suppression), and for favorable outcome prediction in cases of “benign EEG” features (i.e.,no malignant or highly malignant patterns) [9, 10]. However, studies focusing on the prognostic value of EEG patterns in severe stroke requiring MV remains scarce [6, 11].

In the present study, we aimed to investigate the prevalence and the prognostic value of early EEG abnormalities in adult stroke patients requiring MV and ICU admission.

## Methods

### Population

SPICE EEG is a pre-planned, ancillary study of the prospective multicenter longitudinal cohort “SPICE” study, which assessed functional outcomes at one year after stroke requiring MV in the ICU [3]. The study protocol was published previously [2], approved by the Comité de Protection des Personnes Sud Méditerranée 1 (ID RCB 2017-A02452-51) and registered in Clinical Trials (NCT03335995). The SPICE study enrolled adult patients with any type of stroke (i.e., ischemic stroke, intra cerebral hemorrhage, or subarachnoid hemorrhage, excluding those of traumatic origin) and requiring invasive MV in the ICU; Thus, all patients fulfilling the following criteria were eligible for inclusion in SPICE EEG: (1) aged 18 years or more; (2) an acute stroke (i.e., ischemic stroke, intracerebral hemorrhage, or subarachnoid hemorrhage) diagnosed on neuroimaging; (3) an ICU admission within seven days before or after stroke onset; (4) the need for invasive MV for at least 24 h; (5) and at least one EEG recording during the ICU stay. Patients were excluded if they met any of the following criteria: (1) stroke of traumatic origin; (2) refusal to participate; (3) privation of liberty by administrative or judicial decision. Patients were recruited at ICU admission from 33 ICUs located in 26 sites (13 university and 13 general hospitals), in the Greater Paris area, France. For each included patient, written information was given by the local investigator and consent was obtained from the patient herself/himself or her/his next-of-kin. If patients regained capacity during ICU stay or at one of the follow-up visits, they were asked to provide informed consent for the use of acute data and follow-up. Data were collected in an electronic case report form (eCRF) managed by ICUREsearch (ICUREsearch, Paris, France), as previously described [2]. The study was conducted according to the STROBE guidelines [12].

### Data collection

Data prospectively collected by local investigators on eCRF during ICU stay included epidemiological data (age and gender), data on initial stroke management (stroke subtype, GCS and NIHSS score at diagnosis), ICU admission characteristics (main reason for ICU admission and SOFA scores with non-neurological SOFA), and ICU outcomes (clinical seizure and duration of ICU stay). During ICU stay, data on vital status, antiseizures medications use, causes of death and decisions to withdraw life sustaining therapies (WLST) were prospectively collected.

### EEG assessment

Intermittent digital EEG with video was recorded for at least 20 to 30 min by a technician using 12–19 scalp electrodes according to center practices [13], positioned according to the standard 10–20 system placement. We collected the first EEG performed after stroke. EEG was reformatted to both bipolar and off-head referential montages, with band-pass filter of 0.53 Hz and 70 Hz and amplification set at 100 µV/cm. According to French recommendations [13], repetitive bilateral auditory (hand claps, patient’s name call) and nociceptive (pressure on the nail) stimulations were systematically performed. EEG indications were left to the discretion of treating physicians. According to recommendations, EEG is usually performed for detection of non-convulsive seizures/status epilepticus, brain death diagnosis or prognostication of outcome in comatose patients [13, 14]. For the current study, the EEG was routinely interpreted by local expert neurophysiologists (NK, EPR, VB, JPL, LN, and EA). EEG findings were prospectively collected from the daily report of neurophysiologists in the medical record of each included patient. For each patient, the following EEG features were assessed and prospectively collected in the eCRF (Additional file 1: Table 1): background continuity and symmetry, background reactivity to auditory and/or nociceptive stimulation, presence of generalized or localized periodic discharges, presence of triphasic waves and electrographic seizures. EEG reactivity to stimulation was defined as a reproducible modification of EEG background, either an increase or a decrease in amplitude or frequency according to the ACNS terminology [15].

We used a simplified version of the Westhall et al. classification (developed in CA patients [16]), describing EEG patterns as “highly malignant”, “malignant” or “benign”. This classification was made by two neurointensivists with EEG expertise (SB and RS), who were blinded to other clinical data. A highly malignant pattern was defined as suppression, suppression with periodic discharges, or burst-suppression. A malignant pattern was defined as periodic or rhythmic patterns (including electrographic seizures), and nonreactive background. [16]. Finally, a benign EEG was defined in the absence of malignant or highly malignant features, i.e., when all of the 5 following criteria were present: (1) reactivity to auditory and pain stimulations, (2) absence of electrographic seizures, (3) no periodic discharges and (4) no bust suppression patterns, (5) no triphasic waves.

### ICU management

Neurological management was left to the discretion of investigators in the different participating centers. Sedative drug (i.e., propofol and/or midazolam) prescriptions were left to the discretion of investigators and used in accordance with current recommendations [17]. Standard of care for general supportive measures included head elevation (≥ 30°), oxygenation targets > 94%, correction of hypotension, correction of hyperthermia (temperature > 38 °C), correction of hypoglycemia (< 60 mg/dL), and deep vein thrombosis prophylaxis, in accordance with stroke guidelines [18, 19]. Clinical status epilepticus was defined as a seizure whose motor manifestations extend beyond five minutes or by seizure repetition (≥ 2) during short intervals, without interictal consciousness [20]. Prophylactic antiseizure medications (ASMs) were not systematically prescribed after stroke, in accordance with guidelines [19]. In cases of clinical or electrographic seizure, the choice of ASMs was left to the discretion of the physician.

### Outcomes

Outcomes at one year after ICU admission (vital status, modified Rankin scale mRS) were assessed via telephone interviews by an independent research assistant trained for neurological evaluation and scoring on the mRS, and blinded to clinical data at admission and EEG results [21]. When patients were unable to be evaluated directly, functional evaluation was performed with help of family members or professional caregivers, as appropriate. The primary endpoint was functional outcome at one year on the mRS, categorized as unfavorable (mRS 4–6, indicating severe disability or death) or favorable (mRS 0–3). Causes of death were categorized by investigators into two groups, i.e. neurologic (brain death or withdrawal of life sustaining therapies (WLST)) or systemic causes (cardiovascular events, shock or multiorgan failure).

### Statistical analysis

We reported continuous variables as medians (interquartile range (IQR)) and categorical variables as frequencies (percentage). For between-group comparisons, we used the Wilcoxon rank-sum test for continuous variables and either Pearson’s chi-square test or Fisher’s exact test, as appropriate for categorical variables. Clinically relevant non-collinear variables associated with the primary endpoint (*p* value < 0.10) in univariable analysis were entered into the multivariable model. As ‘reactivity to auditory stimulation' and ‘reactivity to painful stimulation' variables were collinear with 'benign EEG', only 'unreactive EEG' variables were included in the multivariable model. The multivariable analysis was adjusted for three a priori defined clinical confounders (i.e., stroke sub-type, sedation at time of EEG recording and delay between stroke and EEG). Log-linearity of continuous variables included in the final model was tested. The log-linearity of quantitative variables included in the multivariate analysis was verified using an additive model with a univariate smoothing spline. In the event of non-log-linearity, quantitative variables were categorized based on their distribution (quartiles and median). Missing values of independent variables were imputed to the median or mode for continuous and categorical variables, respectively. For all EEG variables independently associated with outcome, we calculated sensitivities, specificities, positive predictive values and negative predictive values, with their 95% confidence intervals. All tests were two-sided, and a p-value < 0.05 was considered significant. All statistical analyses were performed using SAS 9.4.

## Results

### Patients’ characteristics

Between March 7, 2017, and December 26, 2019, 364 patients were enrolled in SPICE, among which 153 patients (42%) had had at least one EEG recording during the ICU stay (Additional file 1: Fig. 1) and were included in SPICE EEG. Compared to patients without EEG, patients included in SPICE EEG presented more frequent clinical seizure events at ICU admission (6.5 vs 0.9%), more frequent anti-epileptic drug prescriptions during ICU stay (47.7 vs 10.2%) and a higher NIHSS score (20 (IQR 8–28) vs 16 (IQR 6.5–23), *p* = 0.015) (Additional file 1: Table 2).

Demographic characteristics of the 153 patients included in SPICE EEG are presented in Table 1. Patients had a median age of 62 years (IQR 50–73) and 78/153 (51%) were females. Patients had severe strokes, as reflected by a median GCS score of 10 (IQR 6–14) and a median score on the NIHSS of 20 (IQR 8–28) at diagnosis. Stroke subtypes consisted of 49 ischemic strokes, 52 intracranial hemorrhages, and 52 subarachnoid hemorrhages, including 7 with multiple stroke type. The main indication for initiation of MV was altered mental status (n = 107, 69.9%) and 10 patients (n = 10, 6.5%) presented clinical seizures or status epilepticus at the time of ICU admission. 72/151 (47.7%) patients were treated with ASMs during ICU stay. Compared to favorable outcome patients, patients with unfavorable outcome were older (65.5 vs 56 years, *p* = 0.006), had a lower GCS score (9 vs 11, *p* = 0.02), a higher NIHSS score (NIHSS > 16 in 56/80 (70%) vs 18/39 (42.6%), *p* = 0.012), and a higher non-neurological SOFA score (4 vs 2, *p* = 0.01) score at ICU admission. Finally, patients with an unfavorable outcome were less frequently treated with ASMs (43/107 patients, 40.2%) compared to patients with a favorable outcome (29/44 patients, 65.9%, *p* = 0.004).

**Table 1:** Baseline characteristics and EEG findings

Variables	All patients (N = 153)	Favorable outcome (N = 45)	Unfavorable outcome (N = 108)	*P* value
*Baseline characteristics*				
Age, years	62 [50–73]	56 [48–64]	65.5 [54–76]	0.006
Age ≥ 70 years	51 (33.3)	8 (17.8)	43 (39.8)	0.008
Female gender	78 (51)	24 (53.3)	54 (50)	0.70
*Pre-morbid mRS*				0.32
0	120 (78.9)	40 (88.9)	80 (74.8)	
1	9 (5.9)	3 (6.7)	6 (5.6)	
2	10 (6.6)	2 (4.4)	8 (7.5)	
3	6 (3.9)	0 (0)	6 (5.6)	
4	5 (3.3)	0 (0)	5 (4.7)	
5	2 (1.3)	0 (0)	2 (1.9)	
Charlson comorbidity index ≥ 2	81/151 (53.6)	20/44 (45.5)	61/107 (57)	0.19
*Stroke subtype*				0.09
Intracerebral hemorrhage	52 (34)	11 (24.4)	41 (38)	
Subarachnoid hemorrhage	52 (34)	21 (46.7)	31 (28.7)	
Ischemic stroke	49 (32)	13 (28.9)	36 (33.3)	
*GCS at ICU admission*				
Score	10 [6–14]	11 [8–14]	9 [6–12]	0.02
GCS < 8, indicating coma	92 (60.1)	19 (42.2)	73 (67.6)	0.003
Score on the NIHSS > 16	74/119 (62.2)	18/39 (46.2)	56/80 (70)	0.012
*Reason for ICU admission*				0.18
Altered mental status	107 (69.9)	30 (66.7)	77 (71.3)	
Endovascular therapy or neurosurgery	17 (11.1)	9 (20)	8 (7.4)	
Acute respiratory failure	16 (10.5)	3 (6.7)	13 (12)	
Seizure/status epilepticus	10 (6.5)	3 (6.7)	7 (6.5)	
Shock	3 (2)	0	3 (2.8)	
Non-neurologic SOFA score	3 [1–5]	2 [0–4.5]	4 [2–6]	0.01
EEG findings*				
Delay between stroke and EEG, days	4 [2–7]	4 [3–7]	3 [2–7]	0.38
Sedation during EEG recording	53/151(35.1)	21 (47.7)	32 (29.9)	0.04
Diffuse slowing background	97/150 (64.7)	30/43 (69.8)	67/107 (62.6)	0.38
Focal slowing background	51/151 (33.8)	17/44 (38.6)	34/107 (31.8)	0.16
Periodic discharges	5/136 (3.3)	1/40 (2.3)	4/96 (3.7)	0.9
Triphasic waves	13/137 (8.7)	5/40 (11.6)	8/97 (7.5)	0.76
Electrographic seizure	6/150 (4)	2/43 (4.5)	4/107 (3.7)	0.42
Burst suppression	6/147 (4)	2/43 (4.5)	4/104 (3.7)	1
Unreactive background to auditory and pain stimulations	90/138 (58.8)	19/42 (42.2)	71/96 (65.7)	0.003
Benign EEG**	28/138 (18.9)	16/43 (37.2)	12/105 (11.4)	< 0.001

Results reported as n (%) for categorical variables and median [interquartile range] for continuous variables

EEG: electroencephalogram; GCS: Glasgow coma scale; ICU: intensive care unit; NIHSS National Institutes of Health Stroke Scale SOFA: Sepsis-related organ failure assessment

*The denominator differs in the case of missing data

**A benign EEG is defined when all the five following criteria are present: (1) reactivity to noise and pain, (2) absence of electrographic seizures, (3) no periodic discharges and (4) no burst suppression patterns, (5) no triphasic waves

The median time between stroke and EEG recording was 4 (IQR 2–7) days (Table 1) and 96% of EEGs were performed during ongoing invasive MV. Diffuse and focal slowing were the most frequent abnormalities, observed in 97/150 (64.7%) and 51/151 (33.8%) patients, respectively. In contrast, triphasic waves (13/137, 8.7%), electrographic seizures (6/150, 4%), periodic discharges (5/136, 3.3%) and burst suppression (6/147, 4%) were rarely observed. An unreactive EEG background to painful and auditory stimuli was observed in 90/138 patients (58.8%), and a benign pattern was highlighted in 28/138 patients (18.4%).

### Outcomes

**Fig. 1:**
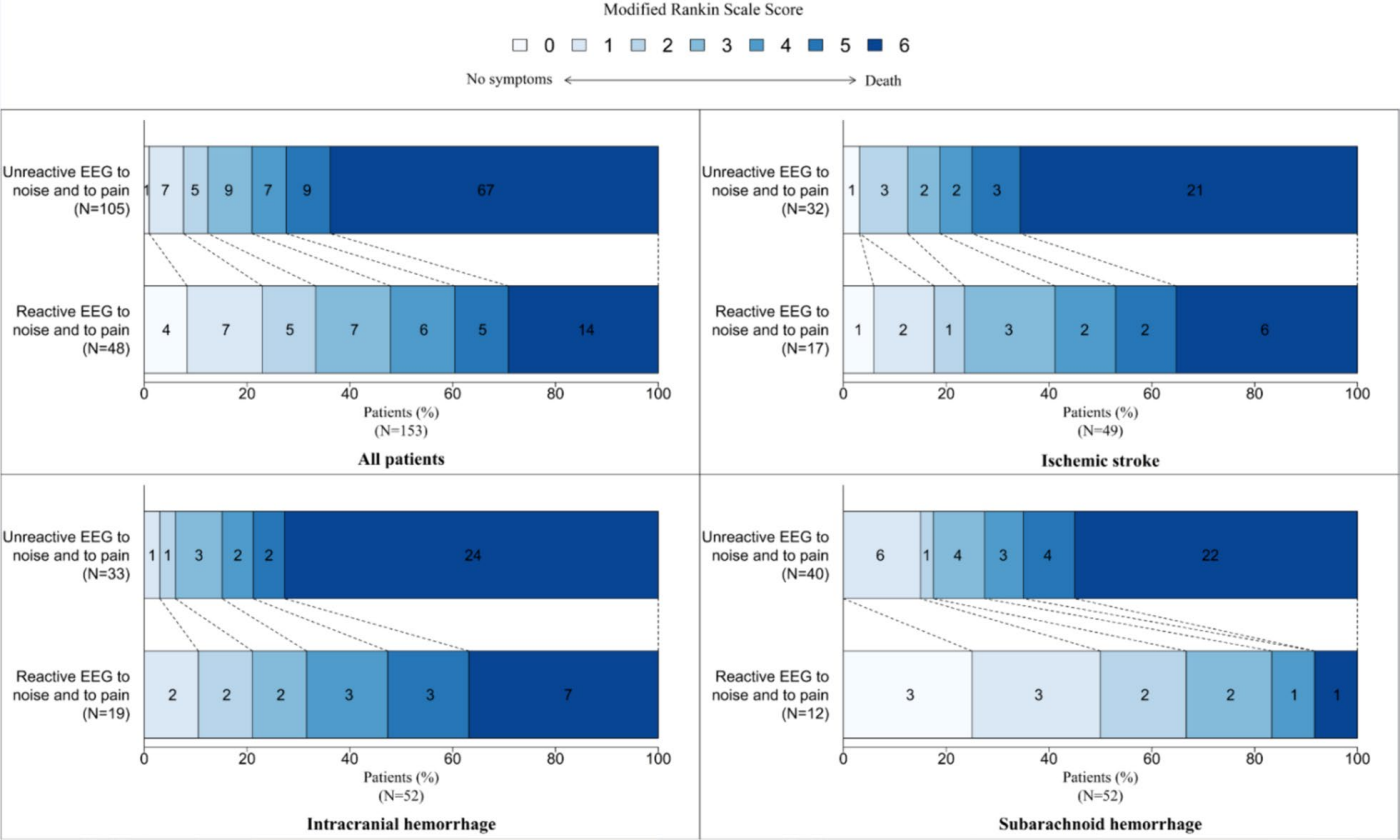
One-year outcomes associated with EEG unreactive to auditory and pain stimulations. The modified Rankin scale (mRS) score is presented in shades of blue

**Fig. 2:**
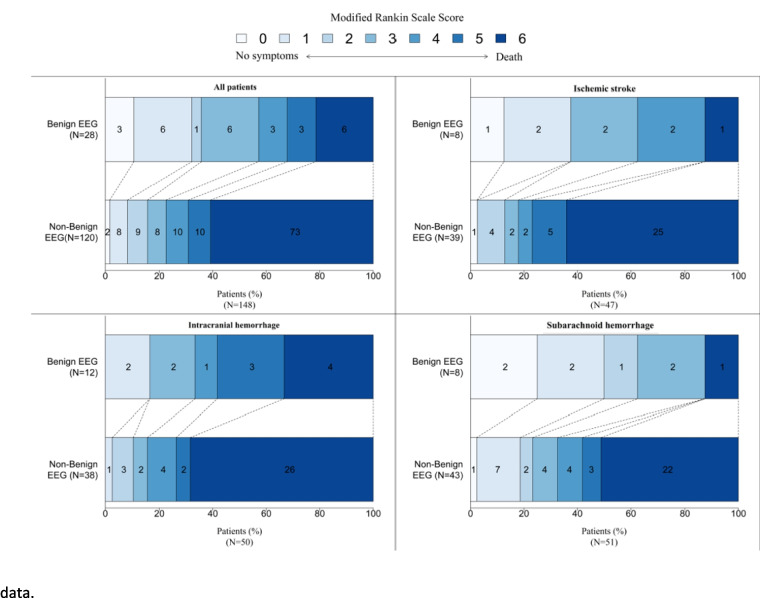
One-year outcome associated with a benign EEG pattern. The modified Rankin scale (mRS) score is presented in shades of blue. A benign EEG is defined when all the 5 following criteria are present: (1) reactivity to auditory and pain stimulations, (2) absence of seizures, (3) no periodic discharges and (4) no burst suppression patterns, (5) no triphasic waves. *n = 148 due to 5 missing data

At one year, 108/153 patients (70%) had an unfavorable outcome, including 81 deaths. Causes of death were related to neurologic causes in 55/81 (69.6%) patients, to systemic causes in 22/81 (27.8%), and unknown in 2/81 (2.5%). An unreactive EEG background to painful and auditory stimuli was more frequently observed in unfavorable outcome patients, as compared to favorable outcome patients (65.7% vs 42.2% respectively, *p* = 0.003) (Fig. 1). Conversely, a benign EEG was more frequently observed among patients with favorable outcome (37.2% vs 11.4%, *p* < 0.001) (Fig. 2). Diffuse background slowing (69.8% vs 62.6%, *p* = 0.38), focal background slowing (38.6% vs 31.8%, *p* = 0.16), periodic discharges (2.3% vs 3.7%, *p* = 0.9), electrographic seizures (4.5% vs 3.7%, *p* = 0.4) or burst suppression (4.5% vs 3.7%, *p* = 1) were not associated with the outcome at one year (Table 1).

### Univariable logistic regression analysis

Univariate logistic regression identified that age (OR 1.03, 95%CI 1–1.06), GCS score < 8 at admission (OR 3.01, 95%CI 1.41–6.41), non-neurological SOFA score (OR 1.19, 95%CI 1.03–1.37), background EEG unreactive to auditory stimulation (OR 4.8, 95%CI 1.84–12.53), EEG unreactive to painful stimulation (OR 4.76 95%CI 1.89–11.96) and EEG unreactive to both auditory and painful stimulations (OR 4.74 95%CI 2.02–11.14) were associated with unfavorable outcome (Additional file 1: Table 3). In contrast, a benign EEG was associated with favorable outcome (OR 0.17, 95%CI 0.06–0.45).

### Multivariable logistic regression analysis

Multivariable analysis adjusted for potential clinical confounders (i.e., age, non-neurological SOFA, GCS < 8 at admission, stroke sub-type and sedation) identified that age (OR 1.05, 95%CI 1.01–1.09), non-neurological SOFA score (OR 1.21, 95%CI 1.02–1.42), GCS < 8 at admission (OR 3.74, 95%CI 1.44–9.72), intracerebral hemorrhage (OR 4.53, 95%CI 1.41–14.57) and an EEG unreactive to auditory and painful stimulations (OR 6.02, 95%CI 2.27–15.99) remained independently associated with unfavorable outcome at one year (Table 2). Conversely, diffuse, or focal slowing background and sedation during EEG recording were not associated with the neurological outcome.

**Table 2:** Prediction of unfavorable outcome at one year, multivariable logistic regression

Variable	Odds Ratio	[95% CI]	*P* value
Age	1.05	[1.01–1.09]	0.008
Non-neurologic SOFA score	1.21	[1.02–1.42]	0.02
GCS < 8 at admission	3.74	[1.44–9.72]	0.007
*Stroke subtype*			
Subarachnoid hemorrhage	1		0.04
Intracerebral hemorrhage	4.53	[1.41–14.57]	
Ischemic stroke	1.95	[0.62–6.17]	
Diffuse slowing background	0.55	[0.19–1.57]	0.26
Focal slowing background	0.91	[0.36–2.29]	0.85
Unreactive EEG to auditory and pain stimulations	6.02	[2.27–15.99]	< 0.001
Sedation during EEG recoding	0.47	[0.19–1.2]	0.11
Delay between stroke and EEG, days	1.02	[0.96–1.09]	0.534

AUC of the model: 0.86 [0.80–0.92]

CI: confidence interval, EEG Electroencephalography, GCS: Glasgow Coma Scale, SOFA: Sepsis-related Organ Failure Assessment

### Performance metrics of EEG reactivity

EEG unreactive to auditory and painful stimulations predicted unfavorable outcome with a specificity of 0.48 (95% CI 0.40–0.56), a sensitivity of 0.79 (95% CI 0.72–0.85), a positive predictive value (PPV) of 0.74 (95% CI 0.67–0.81) and a negative predictive value (NPV) of 0.55 (95% CI 0.47–0.63). Conversely, a benign EEG predicted favorable outcome with a specificity of 0.89 (95% CI 0.84–0.94), a sensitivity of 0.37 (95% CI 0.30–0.45), a PPV of 0.57 (95% CI 0.49–0.65) and a NPV of 0.78 (95% CI 0.71–0.84) **(**Table 3**)**.

**Table 3:** Prognostic values of unreactive and benign EEG

Variable	Prediction	Sensitivity [95% CI]	Specificity [95% CI]	Positive predictive value (PPV) [95% CI]	Negative predictive value (NPV) [95% CI]	TP	TN	FP	FN
Unreactive EEG to auditory and pain stimulations	Poor outcome (mRS 4–6)	0.79 [0.72–0.85]	0.48 [0.40–0.56]	0.74 [0.67–0.81]	0.55 [0.47–0.63]	71	23	25	19
Benign EEG	Good outcome (mRS 0–3)	0.37 [0.30–0.45]	0.89 [0.84–0.94]	0.57 [0.49–0.65]	0.78 [0.71–0.84]	16	93	12	27

mRS: modified Rankin Scale; EEG: electroencephalography; TP: True Positive; TN: True Negative; FP: False Positive; FN: False Negative

The prognostic value of variables associated with poor neurological outcome in the original SPICE study (GCS < 8, NIHSS > 16, age ≥ 70 years and Charlson comorbidity index ≥ 2) are presented in the Additional file 1: Table 4. Compared to unreactive EEG, all of these variables presented lower specificities and lower PPV for poor outcome prediction.

## Effects of sedation on EEG recordings

A total 53/151 (35.1%) patients were sedated during EEG recording **(**Table 1**).** To assess the potential effect of sedation on EEG, we compared the demographic characteristics and EEG findings in patients with and without sedative drug infusion during EEG recording (Additional file 1: Table 5). Clinical seizure at ICU admission and burst suppression were more frequently observed in sedated patients, as compared to non-sedated patients (15.1% vs 2%, *p* = 0.04, and 9.4% vs 1%, *p* = 0.02, respectively). Conversely, electrographic seizures (6.1 vs 0%, *p* = 0.05) and poor outcome at one year (76 vs 60.4%, *p* = 0.04) were more frequent in the non-sedated group. We observed no significant difference regarding unreactive (58% vs 60.4%, *p* = 0.79) or benign pattern (18.8% vs 19.2%, *p* = 0.94) rates between non-sedated and sedated patients.

## Discussion

In this prospective multicenter study conducted in severe stroke patients that required invasive MV, we found that an early background EEG unreactive to painful and auditory stimuli was the only EEG parameter independently associated with unfavorable outcome at one year. This association persisted after adjustment for common clinical confounders, including age, stroke subtype, neurological severity at stroke onset, non-neurological organ failure, and sedation infusion during EEG recording. In contrast, a benign EEG (i.e., reactive and continuous without seizures, periodic discharges, triphasic waves, and burst suppression) was independently associated with favorable outcome at one year.

These results suggest that EEG reactivity is a powerful marker of prognostication in this population of severe stroke patients, compared to other early resting state EEG abnormalities. These results are in line with previous studies conducted in less severe stroke unit patients. In a study conducted in massive cerebral hemispheric infarction patients, a dominant alpha unreactive background was associated with an unfavorable outcome [22]. EEG reactivity reflects dynamic brain responses to external stimulations which requires functional integrity of different neuro-anatomical structures, from the brainstem to the subcortical and cortical areas [23]. In animal models, preserved EEG reactivity is associated with structural and functional integrity of both the cortico-thalamic and the thalamus-brainstem loops, which are key areas involved in consciousness [24]. Moreover, our results suggested that sedation seems to have a limited effect on EEG reactivity. Sedative drugs could modify the EEG spectrum, decreasing frequency and amplitude background. Nevertheless, sedation infusion during EEG recording in CA patients did not modify the prognostic value of unreactive EEG [25]. More and more studies also suggest that light-to-moderate sedation in CA patients does probably not significantly impair the prognostic accuracy of the EEG [26–29]. These results could be explained by a major and recent modification of sedation practices in the ICU, using light to moderate doses, with short acting drugs and a daily interruption of sedation in critically ill patients [30–32]. Nevertheless, these results should be confirmed in a larger cohort of severe strokes of different subtypes.

Although EEG was an independent marker of poor outcome in this population, its prognostic value for unfavorable outcome prediction remained limited. In contrast, a benign EEG accurately predicted favorable outcome. These results were not unexpected, because the prognostic value of EEG reactivity remains also limited in other types of brain injury. Among comatose patients after CA, an unreactive background predicts unfavorable outcome with false positive rates ranging from 0 to 50% [25, 29]. In contrast, a reactive EEG predicts favorable outcome with a PPV between 57 and 85% according to various studies [33]. EEG reactivity could also be useful in traumatic brain injury and more generally among patients with severe acute encephalopathy, but its prognostic performance remains even lower in these populations than in CA patients [5, 34–36]. In short, our results suggested that a benign EEG could reflect limited or reversible brain injury in this population of MV patients with stroke and could thus encourage intensivists to continue aggressive life support. Conversely, an unreactive background likely reflects severe brain injury. Despite this, EEG reactivity should not be used alone for decisions of WLST, due to its low specificity. Ideally, this dynamic neurophysiological marker should be integrated in a multimodal approach together with other robust validated clinical and imaging prognostic tools or biomarkers of brain injury (Neurone-Specific-Enolase or S100b proteins, for example).

Our results also highlighted that diffuse or focal slowing represented the most frequent spot EEG abnormalities, whereas triphasic waves, periodic discharges, and burst suppression were rarely observed during EEG recording. Because EEG patterns may significantly evolve over time, we cannot exclude that these abnormalities were not observed later or earlier during ICU stay. Furthermore, we found no significant association between any of these EEG patterns and one-year outcome. These results are concordant with a previous study in this population which found a significant association between interictal epileptiform discharges and neurological outcome, but no prognostic value of periodic patterns [6]. However, some other studies highlighted that slow background, asymmetry and periodic discharges were independently associated with unfavorable outcome in non-ICU stroke patients [37–40]. These discrepancies could be explained by different factors. First, most of these studies were conducted in stroke unit patients while we only included the most severe stroke cases that required MV at the acute phase. Second, these studies were mainly conducted in ischemic stroke patients [6, 22, 37, 41], whereas SPICE included ischemic and hemorrhagic stroke but also subarachnoid hemorrhage [3]. Third, slowing background, periodic discharges and/or triphasic waves are common in ICU patients with encephalopathies of septic, metabolic or toxic origin [34, 42, 43]. Therefore, one could suggest that these EEG abnormalities observed in our population are multifactorial and non-specific to stroke severity itself. Fourth, periodic discharges are often associated with seizures, which in turn contributes to secondary brain injury and worse outcomes. However, these patterns were relatively infrequent in our study [38]. Moreover, the absence of impact of such EEG patterns on outcome could simply be due to a lack of power. Our study also identified that 6.5% of patients had electrographic seizures during EEG. Interestingly, most seizures occurred during the first seven days after stroke onset, meeting the definition of acute symptomatic seizure [44]. These electrographic seizures could be related to the ischemic brain damage itself or be secondary to intracranial hypertension in the most severe patients [45]. We cannot exclude that this low prevalence was related to the use of intermittent EEG recording, and that this prevalence could have been higher in patients monitored with continuous EEG[46]. Nevertheless, this result is relatively concordant with those of others studies, mainly conducted in non-ICU ischemic stroke patients [6, 47–49].

Our study has several strengths, including its “real life” situation, with a large multicenter recruitment of ICU patients, and prospective collected data. We a priori defined outcomes at one year, which were measured by an independent trained research assistant. EEGs were prospectively analyzed and collected based on the critical care ACNS terminology, limiting the risk of heterogeneity [50]. Finally, we performed all analyses stratified on centers and adjusted for common confounding factors, including sedation at time of EEG recording.

This study also has limitations. First, we did not re-analyze EEG recordings for study purposes. Second, we assessed EEG reactivity with a visual analysis. Because EEG reactivity assessment is subjective with a moderate inter-rater agreement between neurophysiologists [51], automated quantitative approaches have been developed [52]. Nevertheless, these technics are not used in routine practice. Third, there could be significant variability in EEG reactivity assessment among centers, especially regarding the type of stimuli used [23]. In our real-life study, the reactivity testing protocol was not standardized across different centers, although participants were asked to follow French recommendations regarding the use of EEG in the ICU [13]. Fourth, physicians in charge of included patients were not blinded to EEG results. Consequently, we cannot exclude that this may have resulted in self-fulfilling prophecy for WLST decisions during ICU stay. Nevertheless, EEG reactivity is not included in the guidelines algorithm of neuroprognostication for unfavorable outcome prediction, limiting the risk of self-fulfilling prophecy. Although we did not collect specific indications of EEG studies, we speculate that EEG recording was performed for persistent altered mental status, seizure suspicion and/or prognostication. Moreover, we only collected the first EEG performed after stroke, and did not use continuous EEG, because this is not the practice in the majority of French ICU centers. Finally, we had no data regarding doses and types of sedative drugs during EEG recordings, and we cannot exclude that reactivity might have been affected by concomitant sedation. However, only 35% of patients were sedated during EEG and we found no difference of EEG reactivity between sedated and non-sedated patients.

## Conclusion

In this multicenter prospective cohort of severe stroke patients that required invasive ventilation in ICU, the absence of EEG reactivity was independently associated with unfavorable outcome at one year. However, its value for prediction of unfavorable outcome remained limited. Conversely, a benign EEG was associated with favorable outcome at one year. These results confirm that EEG could be useful in severe stroke patients, not only for detection of electrographic seizure but also for assessment of brain injury severity and prognostication, integrated in a multimodal approach.

### Supplementary Information

**Additional file 1**. **Table 1.** EEG data collected in the electronic case report form (eCRF) of the SPICE study. **Table 2.** Patient characteristics according to EEG recoding. **Table 3.** Univariable logistic regression for prediction of unfavorable outcome at one year. **Table 4.** Prognostic values of GCS<8, NIHSS>16, Age ≥ 70 years and Charlson comorbidity index ≥2 for poor outcome prediction. **Table 5.** Demographic characteristics and EEG findings according to sedation during EEG recording. **Figure 1.** Flow chart.

## Data Availability

Data could be available if request to corresponding author, to a reasonable extent.

## Abbreviations

ACNS: American Clinical Neurophysiology Society
ASMs: Antiseizure medications
CA: Cardiac Arrest
CI: Confidence Interval
eCRF: Electronic case report form
EEG: Electroencephalogram
ESM: Electronic Supplementary Material
GCS: Glasgow Coma Scale
HIBI: Hypoxic Ischemic Brain Injury
ICU: Intensive Care Unit
IQR: Inter Quartile Range
MV: Mechanical Ventilation
mRS: Modified Rankin Scale
NIHSS: National Institutes of Health Stroke Scale
OR: Odds Ratio
SOFA: Sequential Organ Failure Assessment
TBI: Traumatic Brain Injury
WLST: Withdrawal of Life Sustaining Therapies
