# Supplementary material for: Prognostic value of early EEG abnormalities in severe stroke patients requiring mechanical ventilation: a pre-planned analysis of the SPICE prospective multicenter study
Source: Crit Care. 2024 May 23;28:173. doi: 10.1186/s13054-024-04957-5 (PMC11119574; doi:10.1186/s13054-024-04957-5)
Supplement: Supplementary file 1 — Additional file 1. Table 1. EEG data collected in the electronic case report form (eCRF) of the SPICE study. Table 2. Patient characteristics according to EEG recoding. Table 3. Univariable logistic regression for prediction of unfavorable outcome at one year. Table 4. Prognostic values of GCS<8, NIHSS>16, Age ≥ 70 years and Charlson comorbidity index ≥2 for poor outcome prediction. Table 5. Demographic characteristics and EEG findings according to sedation during EEG recording. Figure 1. Flow chart. [file 13054_2024_4957_MOESM1_ESM.docx]

**Supplementary material**

| **eCRF SPICE : Stroke Prognosis in Intensive CarE**  Reference  ECRF0012V01  Date : 06/12/2017 |  |  |  |  |  |  |  |  |  |  |  |  |
| --- | --- | --- | --- | --- | --- | --- | --- | --- | --- | --- | --- | --- |
| ECRF0012V01 | Yes    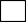 | 1 | No    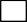 | 2 | I don’t know    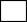 | 9 | _ | _ | Date    / _ _  / _ |  |  | _ |
| **Sedation during EEG recording** | Yes    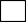 | 1 | No    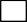 | 2 | I don’t know    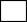 |  | 1 | | | | | |
| **Diffuse slow background** | Yes    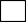 | 1 | No    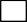 | 2 | I don’t know    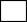 |  | 1 | | | | | |
| **Focal slow background** | Yes    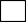 | 1 | No    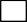 | 2 | I don’t know    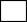 |  | 1 | | | | | |
| Periodic discharges | Yes    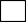 | 1 | No    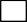 | 2 | I don’t know    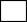 |  | 1 | | | | | |
| **seizures** | Yes    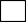 | 1 | No    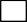 | 2 | I don’t know    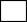 |  | 1 | | | | | |
| **Reactive background to auditory stimulus** | Yes    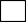 | 1 | No    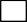 | 2 | I don’t know    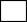 |  | 1 | | | | | |
| **Reactive background to pain** | Yes    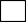 | 1 | No    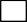 | 2 | I don’t know    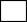 |  | 1 | | | | | |
| **Triphasic waves** | Yes    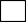 | 1 | No    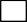 | 2 | I don’t know    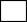 |  | 1 | | | | | |
| **Burst suppression** | Yes    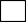 | 1 | No    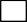 | 2 | I don’t know    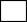 |  | 1 | | | | | |

**Table 1. EEG data collected in the electronic case report form (eCRF) of the SPICE study**.

| **Variables** | **All patients  (N=364)** | **Patients with no EEG (N=211)** | **Patients with EEG  (N=153)** | **P value** |
| --- | --- | --- | --- | --- |
| **Reason for ICU admission** |  |  |  |  |
| Shock | 14 (3.8) | 11 (5.2) | 3 (2) | NS |
| **Clinical seizure** | **12 (3.3)** | **2 (0.9)** | **10 (6.5)** | **0.03** |
| Peri-procedural | 41 (11.3) | 24 (11.4) | 17 (11.1) | NS |
| Respiratory | 37 (10.2) | 21 (10) | 16 (10.5) | NS |
| Impairment of consciousness | 260 (71.4) | 153 (72.5) | 107 (69.9) | NS |
| GCS | **10 [6 ; 14]** | **11 [6 ; 14]** | **10 [6 ; 14]** | **0,12** |
| **NIHSS** | **18 [7 ; 26]** | **16 [6.5 ; 23]** | **20 [8 ; 28]** | **0.015** |
| Stroke subtype |  |  |  | 0.47 |
| Intracerebral hemorrhage | 121 (33.2) | 74 (35.1) | 47 (30.7) | NS |
| Subarachnoid hemorrhage | 103 (28.3) | 53 (25.1) | 50 (32.7) | NS |
| Ischemic stroke | 123 (33.8) | 74 (35.1) | 49 (32) | NS |
| Multiple stroke type | 17 (4.7) | 10 (4.7) | 7 (4.6) | NS |
| **Anti-seizure medication use** | **93 (26.1)** | **21 (10.2)** | **72 (47.7)** | **<.0001** |

**Table 2. Patient characteristics according to EEG recoding. GCS: Glagow Coma Scale. NIHSS: National Institutes of Health Stroke Scale.**

Results reported as n (%) for categorical variables and median [IQR] for continuous variables. NS: non-significant.

| **Variables** | **OR** | **CI [95%]** | **P value** |
| --- | --- | --- | --- |
| ***Baseline characteristics*** |  |  |  |
| **Age** | **1.03** | **[1 ; 1.06]** | **0.04** |
| Stroke sub-type: |  |  | 0.30 |
| Subarachnoid hemorrhage | 1 |  |  |
| Intracerebral hemorrhage | 1.34 | [0.51 ; 3.53] |  |
| Ischemic stroke | 0.66 | [0.27 ; 1.62] |  |
| Delay between stroke and mechanical ventilation | 1.01 | [0.82 ; 1.25] | 0.91 |
| Charlson comorbidity index > or = 2 | 1.69 | [0.79 ; 3.62] | 0.17 |
| Direct admission in ICU | 1.55 | [0.74 ; 3.26] | 0.25 |
| **GCS <8 at ICU admission** | **3.01** | **[1.41 ; 6.41]** | **0.004** |
| **Non-neurological SOFA** | **1.19** | **[1.03 ; 1.37]** | **0.02** |
| ***EEG findings*** |  |  |  |
| Diffuse slow background |  |  |  |
| Yes | 1 |  | 0.85 |
| No | 1.05 | [0.43 ; 2.58] |  |
| Unknown | 0.64 | [0.12 ; 3.38] |  |
| Focal slow background |  |  |  |
| Yes | 1 |  | 0.34 |
| No | 1.28 | [0.57 ; 2.84] |  |
| Unknown | 0.31 | [0.04 ; 2.26] |  |
| Periodic discharges |  |  |  |
| Yes | 1 |  | 0.37 |
| No | 0.51 | [0.05 ; 5.65] |  |
| Unknown | 1.29 | [0.08 ; 20.55] |  |
| **Reactivity to auditory stimulation** |  |  |  |
| Yes | 1 |  | **0.006** |
| No | **4.8** | **[1.84 ; 12.53]** |  |
| Unknown | 3.12 | [0.78 ; 12.5] |  |
| **Reactivity to pain stimulation** |  |  |  |
| Yes | 1 |  | **0.003** |
| **No** | **4.76** | **[1.89 ; 11.96]** |  |
| Unknown | 3.87 | [0.92 ; 16.32] |  |
| Triphasic waves |  |  |  |
| Yes | 1 |  | 0.58 |
| No | 1.93 | [0.54 ; 6.91] |  |
| Unknown | 2.14 | [0.33 ; 13.78] |  |
| Burst suppression |  |  |  |
| Yes | 1 |  | 0.98 |
| No | 1.17 | [0.19 ; 7.33] |  |
| Unknown | 1.08 | [0.05 ; 21.26] |  |
| **Benign EEG** |  |  |  |
| Yes | 1 |  | **0.005** |
| **No** | **3.43** | **[1.44 ; 8.14]** |  |
| Sedation during EEG recording | 0.57 | [0.27 ; 1.22] | 0.15 |

**Table 3. Univariable logistic regression for prediction of unfavorable outcome at one year.** CI: confidence interval; EEG: electroencephalogram; GCS: Glasgow coma scale; OR: odds ratio; SOFA Sepsis-related organ failure assessment.

| **Variable** | **Sensitivity [95%CI]** | **Specificity** | **Positive predictive value (PPV)** | **Negative predictive value (NPV)** | **TP** | **TN** | **FP** | **FN** |
| --- | --- | --- | --- | --- | --- | --- | --- | --- |
|  |  | **[95%CI]** | **[95%CI]** | **[95%CI]** |  |  |  |  |
| **GCS < 8** | 0,79 | 0,43 | 0,68 | 0,58 | 73 | 26 | 35 | 19 |
|  | [0.73 ; 0.86] | [0.35 ; 0.50] | [0.60 ; 0.75] | [0.50 ; 0.66] |  |  |  |  |
| **NIHSS > 16** | 0,76 | 0,47 | 0,70 | 0,54 | 56 | 21 | 24 | 18 |
|  | [0.69 ; 0.82] | [0.39 ; 0.55] | [0.63 ; 0.77] | [0.46 ; 0.62] |  |  |  |  |
| **Age** ≥ **70 years** | 0,84 | 0,36 | 0,40 | 0,82 | 43 | 37 | 65 | 8 |
|  | [0.79 ; 0.90] | [0.29 ; 0.44] | [0.32 ; 0.48] | [0.76 ; 0.88] |  |  |  |  |
| **Charlson comorbidity index** ≥ **2** | 0,75 | 0,34 | 0,57 | 0,55 | 61 | 24 | 46 | 20 |
|  | [0.68 ; 0.82] | [0.27 ; 0.42] | [0.49 ; 0.65] | [0.47 ; 0.62] |  |  |  |  |

**Table 4. Prognostic values of GCS<8, NIHSS>16, Age** ≥ **70 years and Charlson comorbidity index** ≥**2 for poor outcome prediction.** GCS: Glasgow Coma Scale; NIHSS: National Institutes of Health Stroke Scale; TP: True Positive; TN: True Negative; FP: False Positive; FN: False Negative.

| **Variable** | **All**  **patients**  **N=153** | **No sedation during EEG  N=100** | **Sedation during EEG N=53** | **P value** |
| --- | --- | --- | --- | --- |
| ***Baseline characteristics*** |  |  |  |  |
| Age, years | 62 [50 ; 73] | 64 [52 ; 74] | 57 [48 ; 68] | 0.09 |
| Female sex | 78 (51) | 53 (53) | 25 (47.2) | 0.49 |
| Stroke subtype |  |  |  |  |
| Intracerebral hemorrhage | 52 (34) | 34 (34) | 18 (34) | 1 |
| Subarachnoid hemorrhage | 52 (34) | 34 (34) | 18 (34) |  |
| Ischemic stroke | 49 (32) | 32 (32) | 17 (32.1) |  |
| GCS at ICU admission | 10 [6 ; 14] | 10 [6 ; 13] | 10 [6 ; 14] | 0.16 |
| GCS <8 at admission | 92 (60.1) | 61 (61) | 31 (58.5) | 0.76 |
| Reason for ICU admission |  |  |  | 0.04 |
| Altered mental status | 107 (69.9) | 73 (73) | 34 (64.2) |  |
| Endovascular therapy or neurosurgery | 17 (11.1) | 12 (12) | 5 (9.4) |  |
| Acute respiratory failure | 16 (10.5) | 11 (11) | 5 (9.4) |  |
| Clinical seizure | 10 (6.5) | 2 (2) | 8 (15.1) |  |
| Shock | 3 (2) | 2 (2) | 1 (1.9) |  |
| Non-neurologic SOFA score | 3 [1 ; 5] | 3 [1 ; 5] | 3.5 [1 ; 5] | 0.99 |
| ***EEG findings*** |  |  |  |  |
| Time between stroke and EEG, days | 4 [2 ; 7] | 4 [2 ; 8] | 3 [2 ; 5] | 0.08 |
| Diffuse slowing background | 97/150 (64.7) | 61 (62.2) | 36 (69.2) | 0.21 |
| Focal slowing background | 51/151 (33.8) | 33 (33.7) | 18 (34) | 1 |
| Periodic discharges | 5/136 (3.3) | 4 (4.1) | 1 (1.9) | 0.42 |
| Triphasic waves | 13/137 (8.7) | 11 (11.3) | 2 (3.8) | 0.34 |
| **Electrographic seizure or status epilepticus** | **6/150 (4)** | **6 (6.1)** | **0 (0)** | **0.05** |
| **Burst suppression** | **6/147 (4)** | **1 (1)** | **5 (9.4)** | **0.02** |
| Unreactive to auditory and pain stimulation | 90/138 (58.8) | 58 (58) | 32 (60.4) | 0.79 |
| Benign EEG* | 28 (18.9) | 18 (18.8) | 10 (19.2) | 0.94 |
| **Poor outcome at one year** | **108 (70.6)** | **76 (76)** | **32 (60.4)** | **0.04** |

**Table 5. Demographic characteristics and EEG findings according to sedation during EEG recording.** Results reported as n (%) for categorical variables and median [IQR] for continuous variables.

EEG: electroencephalogram; GCS: Glasgow coma scale; ICU: intensive care unit. SOFA Sepsis-related organ failure assessment. *A benign EEG is defined when all of the 5 following criteria are present: 1) reactivity to auditory and pain stimulations, 2) absence of seizure/status epilepticus, 3) no periodic discharges and 4) no burst suppression patterns 5) no triphasic waves.


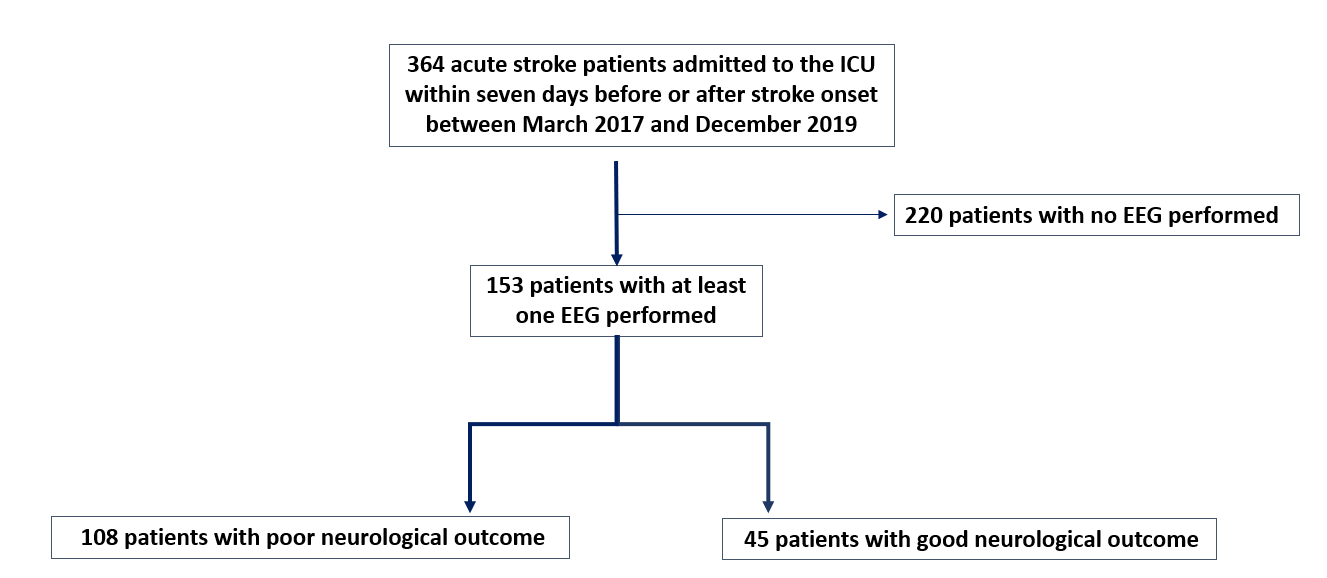


**Figure 1 supplementary data. Flow chart**
